# Visualizing DNA repair factor recruitment at sites of transcription in single cells

**DOI:** 10.1007/s10577-025-09789-9

**Published:** 2026-01-03

**Authors:** Tianyi Guan, Yuepeng Shi, Tae-Hee Lee, Philipp Oberdoerffer

**Affiliations:** 1 Department of Radiation Oncology and Molecular Radiation Sciences, Johns Hopkins University School of Medicine, Baltimore, MD 21287 USA; 2 University of Illinois, Champaign, IL USA

**Keywords:** Topoisomerase 1, Transcription, Immunofluorescent imaging, DNA repair

## Abstract

Transcription-associated torsional stress presents a continuous threat to genome integrity that needs to be tightly controlled. Here we present a method for the visualization of genome maintenance events at sites of active transcription, which builds on a previously developed reporter system that allows for the detection of nascent, MS2-repeat containing transcripts with a YFP-tagged MS2 coat protein. We describe steps to monitor Topoisomerase 1 engagement as a result of transcription-associated topological stress in both S phase and non-S phase cells, and detail procedures to visualize TOP1 cleavage complex formation and concomitant DNA repair factor engagement. We expect this system to provide a powerful means to study effectors of the transcription-associated topological stress response.

**Supplementary Information:**

The online version contains supplementary material available at 10.1007/s10577-025-09789-9.

## Introduction

Transcription requires the unwinding of DNA by RNA polymerase, which causes topological stress, a potential source of genome instability (Pommier et al 2022, 2016). DNA topoisomerase I (TOP1) resolves DNA supercoils by nicking one strand of the DNA double-helix. This process involves the transient formation of a covalent TOP1-DNA cleavage complex (TOP1cc) and adjacent single-stranded DNA (ssDNA) break, followed by supercoil unwinding and re-ligation of the broken DNA (Pommier et al. 2016; Ray Chaudhuri & Nussenzweig 2017). If unresolved, TOP1ccs impede essential DNA transactions and have been linked to somatic mutations, chromosomal aberrations and cell death (Pommier et al. 2022; Reijns et al 2022). The resolution of catalytically trapped TOP1ccs requires specialized TOP1:DNA adduct removal, resulting in a ssDNA gap that is resolved via canonical ssDNA break repair (SSBR) (Caldecott 2022; Ray Chaudhuri & Nussenzweig 2017). Poly(ADP-ribose) (PAR) Polymerase 1 (PARP1) is a multifaceted effector of TOP1cc repair that facilitates TOP1cc degradation, ssDNA end processing and SSBR of the lesion (Fabian et al 2024; Ray Chaudhuri & Nussenzweig 2017; Sun et al 2021). How TOP1cc repair events are orchestrated in the context of transcription-associated topological stress remains poorly understood.

Existing tools to study TOP1cc repair largely rely on the biochemical detection of TOP1cc turnover or imaging-based detection of TOP1 inhibitor-induced DNA lesions (Kiianitsa & Maizels 2014; Kuzin et al 2022; Patel et al 2016). Neither of these approaches provide information that accounts for the genomic context in which a repair event occurs, which is particularly relevant when interrogating the impact of the epigenome on DNA repair outcomes (Chen & Tyler 2022; Dabin et al 2016). TOP1 Covalent Adduct Detection (CAD), a more recently described next generation sequencing (NGS)-based technique, allows for the genome-wide mapping of TOP1ccs, but the analysis of TOP1cc-associated repair events remains correlative, involving parallel mapping of DNA repair factors in a separate cell population (Baranello et al 2016; Kuzin et al. 2022). To address these limitations, we have developed an approach for the imaging-based detection of locally defined TOP1cc repair events (Lee et al 2025). Our approach is conceptually analogous to the study of DNA double-strand breaks (DSBs) at defined locations in single nuclei, which has significantly advanced our understanding of DSB repair (Vitor et al 2020).

**Fig. 1:**
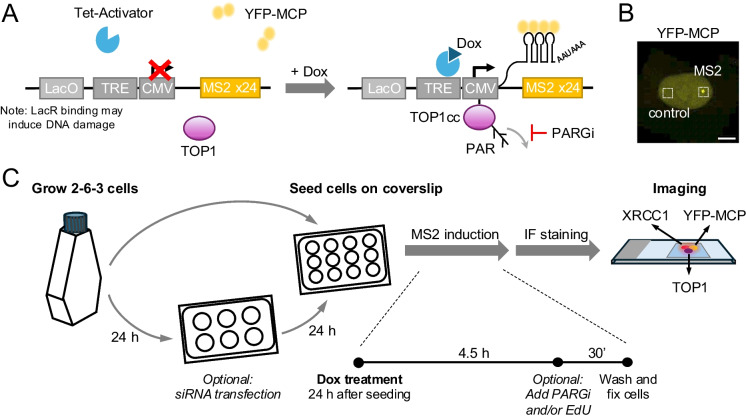
Approach to monitor transcription-associated DNA repair in single cells. **A** Schematic of the MS2 reporter system. U2OS 2-6-3 cells express the Tet activator, a YFP-tagged MS2 coat protein (YFP-MCP) and a Dox-inducible transgene encoding a 24xMS2 mRNA. Dox treatment results in MS2 transgene expression, which is bound by YFP-MCP. PARG inhibitor (PARGi) treatment serves to stabilize PAR chains. An adjacent LacO repeat array can be used to tether proteins of interest fused to the LacI repressor. Note that LacI binding to LacO poses a DNA replication block that triggers the replication stress response and may interfere with TOP1cc-associated DNA repair factor detection. **B** Representative IF image of nuclear YFP-MCP expression following 5 h of Dox treatment. Squares depict the MS2 site or a control region used for downstream analysis. Scale bar: 5 µm (**C**) Wet lab workflow: U2OS 2-6-3 cells are seeded on coverslips in 12 well-plates. If desired, experimental perturbation such as siRNA transfection is performed 24 h prior to seeding. Dox is added for 5 h the day after cells were seeded on coverslips. If desired, PARGi and/or EdU are added to Dox media 4.5 h into Dox treatment for 30 min. Cells are then fixed, stained for proteins of interest and subjected to IF analysis

To monitor TOP1cc repair factor engagement at an actively transcribed genomic locus, we take advantage of a U2OS cell-based transcriptional reporter system that allows for the doxycycline (Dox)-inducible expression of a transgene encoding a transcript with 24 MS2 stem-loop repeats. Nascent mRNA is detected at the site of transcription via yellow fluorescent protein (YFP)-tagged viral MS2 coat protein (YFP-MCP), encoded by a separate transgene (Fig. 1A, B) (Janicki et al 2004; Shanbhag et al 2010; Tang et al 2013). TOP1 Covalent Adduct Detection followed by qPCR revealed robust TOP1cc accumulation immediately flanking the MS2 transcription start site, demonstrating efficient TOP1 activation following Dox-induced MS2 expression (Lee et al. 2025). Here, we describe a detailed protocol for the detection of TOP1, the TOP1cc DNA:protein adduct and the XRCC1 SSBR repair factor at MS2 sites using immunofluorescence (IF)-based imaging. Parallel assessment of DNA replication via EdU labeling allows for analysis of TOP1cc repair events separated by cell cycle stage (Fig. 1C).

Using this tool in combination with other imaging and ‘omics approaches, we recently identified the PAR-binding macro-histone variant macroH2A1.1 as a chromatin component that orchestrates PAR-dependent TOP1cc repair (Lee et al. 2025). We envision that this methodology will facilitate the discovery of novel regulators of a broad range of transcription-related DNA transaction including but not limited to TOP1cc repair.

## Step-by-step methods

See Table S1 for a list of all reagents used in this study.

### Cell lines and drug treatments

Human U2OS 2-6-3 osteosarcoma cells carrying transgenes encoding a 24xMS2 mRNA (p3216PECMS2β) and YFP-MCP (gift from R. Greenberg, University of Pennsylvania) are grown in Dulbecco's modified eagle medium (DMEM, Invitrogen) supplemented with 4.5 g/L D-glucose and 10% FBS (Gemini) at 37 °C with 5% CO_2_. Before each set of experiments, cells are reselected for the integration of MS2 and YFP-MCP transgenes using hygromycin (100 µg/ml) and Geneticin (200–400 µg/ml), respectively. Both antibiotics are added simultaneously for a course of 10 days. Cells are regularly tested for mycoplasma using the Mycoplasma PCR detection kit (Abcam). Doxycycline hyclate (Dox, Sigma), Poly(ADP-ribose) glycohydrolase inhibitor (PARGi) (PDD000172273, Selleckchem) and 5-Ethynyl-2’-deoxyuridine (EdU, Sigma-Aldrich) are added to cell cultures as indicated below.

### Antibodies

Primary antibodies: α-TOP1 (Abcam, Cat#ab109374), α-XRCC1 (Novus: Cat# NBP1-87,154), α-TOP1cc (Sigma-Aldrich: Cat# MABE1084), α-H3K27me3 (Cell Signaling Technology: Cat#9733 T). Secondary Antibodies: goat anti-mouse IgG-Alexa 594/647, goat anti-rabbit IgG-Alexa 594/647 (Invitrogen).

### Step 1. MS2 transgene induction.

-
  1.1 **Dox treatment:** U2OS 2-6-3 cells are grown in 1 ml of culture media on poly-lysine-coated 14 mm coverslips in 12-well plates. Cells are seeded at a density of 200,000 to 250,000 cells per well, expanded overnight, and treated with 2 μ g/ml Dox for 5 h at 37 °C to induce MS2 expression.
  1.2  **PAR chain stabilization:** After 4.5 h of Doxycycline treatment, PARGi is added to Dox-treated wells at a final concentration of 10 µM to stabilize PAR chains. Note that PARGi treatment can be omitted if PAR chain stabilization is not desired.
  1.3 **S phase labeling:** If desired, S phase cells are labelled via the incorporation of the thymidine analog 5-Ethynyl-2′-deoxyuridine (EdU), using the EdU Click-it reaction following the manufacturer’s instructions (Invitrogen). In brief, EdU is added to Dox-treated wells at a final concentration of 10 µM after 4.5 h of Doxycycline treatment. When treating with PARGi, EdU and PARGi are added at the same time. 30 min after PARGi/EdU addition, cells are washed with PBS to terminate the treatment (see schematic in Fig. 1C).

### Step 2. Cell fixation, permeabilization and blocking.

-
  2.1  **Fixation:** 4% Formaldehyde in PBS is added to each well and plates are incubated at room temperature (RT) for 10 minutes in the dark to prevent photobleaching of YFP-MCP. Fixation should not exceed 10 minutes to avoid quenching of the YFP signal. Cover slips are washed in PBS, transferred to a new 12-well plate and washed again to completely remove any residual formaldehyde. Coverslips may be stored in 1 ml PBS overnight at 4 °C in the dark.
  2.2 **Permeabilization:** Coverslips are treated with 1 ml 0.5% Triton-X in PBS for 10 minutes at 4 °C and rinsed once with PBS-T (0.1% Triton-X in PBS).
  2.3  **Enhanced antigen retrieval for immunodetection of TOP1ccs:** Sodium dodecyl sulfate (SDS) treatment is recommended when staining for TOP1cc to facilitate antigen retrieval (Lascaux et al 2024; Patel et al. 2016). Following permeabilization, coverslips are washed in PBS and incubated with 1% SDS in PBS at RT for 5 min. Cells are then washed 5 times with PBS-T at RT, followed by two additional washes with PBS. Note that SDS treatment can interfere with the detection of some antigens.
  2.4  **Blocking of non-specific antibody binding:** Coverslips are incubated in 1 ml 5% Bovine serum albumin (BSA) for 1 h at 37 °C or 10% Fetal Bovine serum (FBS) in PBS for 1 h at RT. Cells are then washed three times with PBS-T for 3 min under gentle shaking. Note that BSA is recommended when staining for TOP1cc.

### Step 3. Immunofluorescence staining.

Prior to the subsequent steps, coverslips are transferred to a humidified, light-shielded chamber.

-
  3.1 **EdU-labeling:** EdU Click-iT™ Plus reaction buffer is prepared following the manufacturer’s instructions (Invitrogen). Note that Click-iT™ Plus buffer is recommended as it uses a copper protectant to preserve fluorescent protein signal. Coverslips are incubated with 50 µL reaction buffer for 30 min at 37 °C and washed 3 times with PBS-T. Note that EdU staining of S phase cells results in strong signal. To avoid bleed through into other channels in our imaging system, we use Alexa Azide 647 (Invitrogen) for EdU detection in the far-red channel (see Step 4).
  3.2 **Primary antibody staining:** Coverslips are stained with primary antibodies of interest in a humidified chamber. We recommend the following dilutions and incubation times for antibodies used in this study: α-TOP1, α-XRCC1, α-H3K27me3: 1:300 (v/v) in 2.5% BSA in PBS or 5% FBS in PBS, 3 hours at RT or overnight at 4 °C; α-TOP1cc: 1:100 (v/v) in 2.5% BSA in PBS, 3 hours at RT or overnight at 4°C. TOP1cc staining may be performed prior to staining with other primary antibodies. Following each antibody staining, coverslips are washed in PBS-T followed by two additional washes with PBS by gently rocking the humidified chamber for 3 minutes on a shaker at RT.
  3.3 **Secondary antibody staining:** Coverslips are stained with fluorophore-conjugated secondary antibodies directed against the primary antibody in a humidified chamber. For antibodies used in this study, we recommend a 1:500 (v/v) dilution in 5% FBS/PBS, or 2.5% BSA/PBS when using α-TOP1cc primary antibody. Samples are incubated for 1 hour at RT and coverslips are washed in PBS-T followed by two additional washes with PBS for 3 minutes on a shaker at RT.
  3.4 **Nuclei detection and mounting:** To ensure that the entire coverslip surface is evenly covered during Hoechst staining, coverslips are transferred to a 12 well-plate and incubated with 1 ml Hoechst 33342 diluted 1:2000 in PBS-T for 5 min at RT. Coverslips are washed once with 1 x PBS and mounted onto glass slides using 7 µl of Prolong Dimond Antifade Mountant (Invitrogen, P36930).

### Step 4: Image acquisition and analysis.

All image analysis steps described here are performed using Fiji ImageJ (v2.16.0). Custom ImageJ macros are available on GitHub (https://github.com/tguan6/MS2_Enrichment_Profiling )

**Fig. 2:**
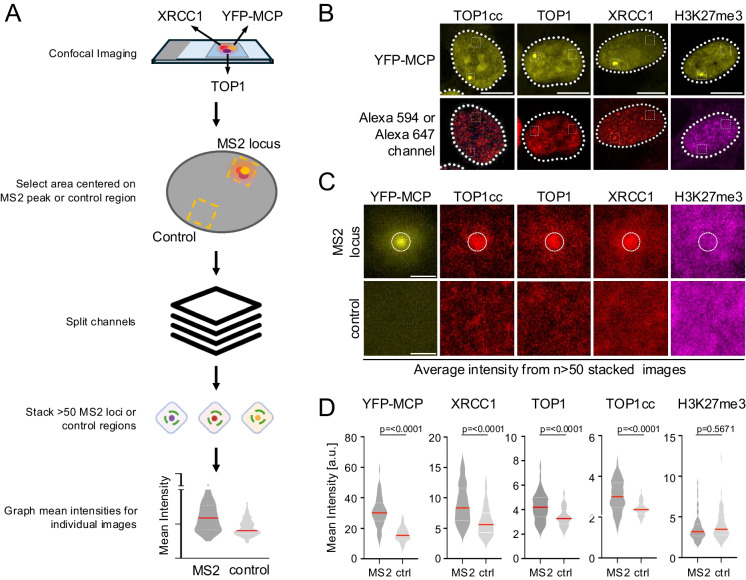
Image-based detection of TOP1-associated DNA damage and repair. **A** Image analysis workflow: Images are acquired on a confocal microscope and analyzed in ImageJ. A 2.87 × 2.87 µm square is centered on the MS2 focus or a nuclear control region of equal size and similar orientation. Images of at least 50 MS2 foci, or the corresponding control regions, are stacked via Z projection to generate single overlay images. For statistical analysis, mean intensities are calculated for each image and channel within a given stack. **B** Representative IF images of nuclei 5 h after Dox-treatment, PARGi was added for 30 min. A corresponding MCP-YFP staining is shown for each protein of interest. Squares depict the MS2 site or a control region used for analyses in (**C**) and (**D**). TOP1cc staining was performed using SDS fixation as described in Methods Section 2.3, scale bars: 10 µm. **C** Z projection overlays as described in (**A**) for the indicated proteins and nuclear regions following Dox induction and PARGi treatment (see Fig. 1C). Scale bar: 1 µm. Displayed images are adjusted in ImageJ using the *Brightness and Contrast function*, with matched minimum/maximum intensity and contrast settings for each MS2/control region pair. Number of images per stack: YFP-MCP (n = 53); TOP1 (n = 50); TOP1cc (n = 54); XRCC1 (n = 53); H3K27me3 (n = 53). **D** Mean intensity distributions of image stacks in (**C**) for areas marked by the white circle (MS2) or the entire 2.87 × 2.87 µm square (control). For all violin plots, center lines (red) represent the median, white dotted lines depict upper and lower quartiles, p values are based on two-sided Mann–Whitney U test for the indicated, pairwise comparisons

-
  4.1 **Image acquisition:** At least 50 MS2 foci-containing nuclei are acquired using a confocal microscope with a 63x oil objective (Numerical Aperture: 1.4). Analyses presented here are from a single focal plane per nucleus, acquired on a Zeiss LSM900 microscope using Zen software version 3.6. The following filter settings were used for each fluorophore:
    Alexa Fluor 405/Hoechst 33342 – excitation: 401 nm; emission: 422 nm
    YFP – excitation: 508 nm; emission: 524 nm
    Alexa Fluor 594 – excitation: 587 nm; emission: 610 nm
    Alexa Fluor 647 – excitation: 653 nm; emission: 668 nm
  4.2 **MS2 foci selection:** For each MS2 focus, a 2.87 x 2.87 µm (58 x 58 pixel) square region of interest (ROI) is manually centered on the MS2 peak such that it stays within the bounds of the nucleus. Optimal ROI size can vary depending on imaging resolution and the protein of interest. A randomly selected MS2-distal nuclear region of equal size serves as a control. Images for each region are saved using the *ROI manager*. Each ROI is processed using a custom ImageJ macro (Foci_Center_Enrichment_Profiling.ijm) to split channels into separate grayscale images, as described in (So et al 2025). Individual MS2 focus or control images are stacked using the *Image sequence* function. Z projections are performed using the *Average Intensity* function for each stack to generate a single overlay image (see schematic in Fig. 2A).

- 4.3
- **MS2 and control region intensity measurements and analysis:** To analyze intensity distributions of a given protein across individual MS2 foci images in a given stack, a 1 μm diameter circle is created in the ‘ROI manager’ and centered on the MS2 peak. The mean MS2 focus intensity for each image within the stack is calculated for each channel using the multi measure function. For control regions, mean intensities are calculated for each 2.87 × 2.87 µm square. If desired, the ratio of MS2 foci and matched control region mean intensities is calculated for each image. Mean intensities or ratio distributions are graphed using Prism or R Studio. To account for non-parametric sample distribution, the two-sided Mann–Whitney U test is used to determine statistically significant differences between two independent sample sets, such as control versus MS2 region, or experimental perturbation versus control treatment.
- ***Note:*** Mean intensity measurements are chosen over maximum intensity for the following reasons: (i) the MS2 locus contains multiple copies of the MS2 transgene. Mean intensity across the 1 µm diameter circle encompassing Dox-induced, nascent MS2 signal is expected to represent nascent mRNA accumulation averaged across all MS2 copies; (ii) mean intensity measurements across the 2.87 x 2.87 µm control region ensure that background signal is not dominated by a strong TOP1, XRCC1 or TOP1cc signal resulting from a single site of endogenous topological stress.

- 4.4
- **Identification of S phase cells**. S phase cells are defined as cells that have incorporated EdU. To identify cells as EdU^+^ or EdU^–^, the average nuclear intensity of Alexa 647 (EdU) is measured for each nucleus. Nuclear ROI segmentation is performed based on Hoechst 33342 signal, and EdU intensity is measured for each ROI using a custom ImageJ macro (Nuclear_Masking_Intensity_Measurement.ijm). The resulting values are normalized to background, based on signal intensity in EdU– control samples. Nuclei with EdU intensity above the control threshold are considered EdU+. MS2 and control region intensity measurements and analyses are performed separately for images from EdU+ and EdU– nuclei.

## Results and discussion

### MS2 induction promotes TOP1cc formation and SSB repair factor accumulation

Dox-induced MS2 expression results in the formation of YFP-MCP foci, which represent nascent pre-mRNA at the site of the MS2 transgene (Fig. 1B). After 5 h, approximately 50% of U2OS 2-6-3 cells show prominent YFP-MCP foci (Janicki et al. 2004; Lee et al. 2025). Transcription-associated recruitment of a protein of interest is expected to result in an accumulation of IF signal at or near the MS2 site. To stabilize repair factor accumulation upon MS2 induction, cells were treated for 30 min with an inhibitor of poly(ADP-ribose) glycohydrolase (PARGi), which prevents the degradation of PAR chains (Sun et al. 2021), a known effector of TOP1cc repair factor recruitment (Caldecott 2019; Ray Chaudhuri & Nussenzweig 2017). To account for cell-to-cell variation and/or weak signal strength at individual foci, we assessed average IF signal intensities for YFP-MCP and the protein of interest across at least 50 YFP-MCP foci per experiment (Fig. 2A). Z projections derived from stacked images at the MS2 site and an MS2-distal control region are shown for YFP-MCP, TOP1, the TOP1cc protein:DNA adduct and the XRCC1 repair factor (Fig. 2B, C). Focal enrichment was observed at the MS2 site but not the control region for each of these proteins, consistent with transcription-induced TOP1cc formation and concomitant XRCC1 engagement. No MS2-associated enrichment was detected for histone H3 trimethylated on lysine 27 (H3K27me3), a repressive histone mark not associated with transcriptional activation (Wiles & Selker 2017) (Fig. 2B, C). The distribution of mean signal intensities derived from each individual MS2 focus or control region in a given stack is graphed as a violin plot in Fig. 2D.

**Fig. 3:**
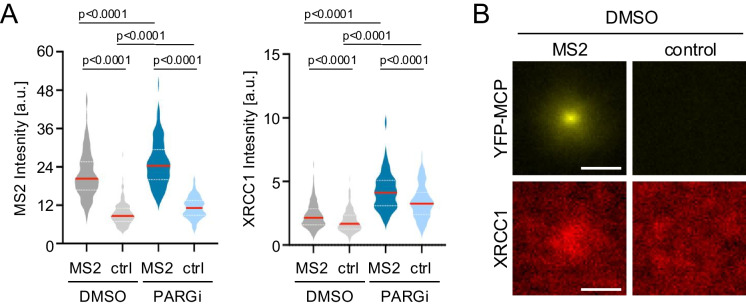
XRCC1 accumulation at the MS2 locus in the absence of PARGi treatment. **A** Mean intensity distributions at MS2 foci or control regions (ctrl) in the presence or absence of PARGi treatment. Number of images per stack: YFP-MCP (DMSO: n = 125; PARGi: n = 122); XRCC1 (DMSO: n = 125; PARGi: n = 122). Center lines (red) represent the median, white dotted lines depict upper and lower quartiles, p values are based on two-sided Mann–Whitney U test for the indicated, pairwise comparisons. **B** Z projection overlays of the indicated proteins at the MS2 or control locus in the absence of PARGi treatment (DMSO) as in Fig. 2C, scale bar: 1 µm. A representative of two independent experiments is shown

Underlining the contribution of PARylation to XRCC1 retention on chromatin and corroborating the rationale for PARGi treatment, XRCC1 signal intensity was significantly reduced in the absence of PAR chain stabilization. Average XRCC1 intensities were decreased both at the control locus and at the MS2 site, indicating reduced overall association of XRCC1 with chromatin (Fig. 3A). We nevertheless observed MS2-specific XRCC1 enrichment in the absence of PARGi (Fig. 3B) and conclude that our system allows for the study of TOP1cc repair both in the presence and absence of PAR chain stabilization. For robust signal detection, we recommend inclusion of PARGi treatment. Taken together, these findings establish the MS2 system as a powerful means to investigate effectors of TOP1cc repair in the context of active transcription in single cells, provided IF-suitable antibodies exist.

### The effect of cell cycle stage on transcription-associated TOP1cc formation

**Fig. 4:**
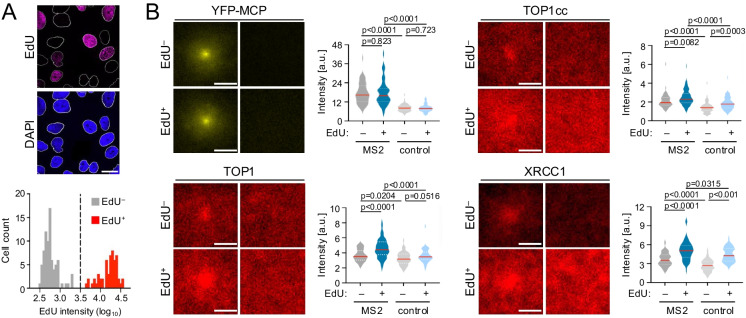
Transcription-associated TOP1 accumulation in S and non-S phase cells. **A** IF analysis of EdU incorporation in U2OS 2-6-3 nuclei, scale bar: 20 µm. The percentage of EdU^+^ and EdU^–^ nuclei is shown for a representative experiment. **B** Z projection overlays and corresponding mean intensity distributions for the indicated proteins at MS2 foci or control regions as described in Fig. 2C, D; scale bar: 1 µm. TOP1cc staining was performed using SDS fixation as described in Methods Section 2.3. Cells were subdivided based on the presence (EdU^+^) or absence of EdU incorporation (EdU^–^). Number of images per stack: YFP-MCP (EdU^+^: n = 118; EdU^–^: n = 128); TOP1 (EdU^+^: n = 54; EdU^–^: n = 56), TOP1cc (EdU^+^: n = 64; EdU^–^: n = 58). For violin plots, center lines (red) represent the median, white dotted lines depict upper and lower quartiles, p values are based on two-sided Mann–Whitney U test for the indicated, pairwise comparisons. A representative of two independent experiments is shown

If unresolved, TOP1ccs pose obstacles to nuclear DNA transactions. During DNA replication in S phase cells, TOP1cc accumulation promotes DNA polymerase stalling, which can cause subsequent DNA break formation and replication stress. In non-S phase cells, TOP1cc formation can impede RNA Polymerase progression, which has been linked to increased RNA:DNA hybrid formation and associated DNA damage (Pommier et al. 2022, 2016). By incorporating EdU labeling into the MS2 workflow, our system allows for the dissection of transcription-associated repair events in S phase versus non-S phase cells (Fig. 4A). We observed similar levels of MS2 transgene induction in EdU^+^ and EdU^–^ cells, supporting the notion that changes observed between the two cell subsets are not a consequence of altered RNA polymerase activity (Fig. 4B). TOP1 and TOP1cc levels were increased both at the MS2 locus and across the control region in S phase cells compared to non-S phase cells, consistent with overall elevated torsional stress in the former, likely as a result of DNA replication (Pommier et al. 2016). XRCC1 levels mirrored the increase in TOP1cc accumulation (Fig. 4B). Despite the difference in overall signal intensity between EdU^–^ and EdU^+^ cells, MS2 loci show significant enrichment of TOP1, TOP1cc and XRCC1 compared to the control locus in both subsets (Fig. 4B). We, therefore, conclude that MS2 induction allows for the monitoring of TOP1 activity and TOP1cc repair in a cell cycle-dependent manner. By focusing downstream analyses on EdU^–^ cells, investigators will be able to dissect potential transcription-associated functions for their gene of interest in the absence of DNA replication as a confounding variable.

### Limitations of the system

Perturbations that interfere with transcription are expected to result in decreased YFP-MCP signal and impaired detection of the nascent MS2 site. For the analysis of fixed samples, as described here, studies are therefore limited to experimental conditions that do not significantly impair MS2 mRNA expression. Alternatively, live cell imaging may be used, wherein a perturbation can be introduced after the MS2 site has been detected. This approach is expected to require additional optimization not described here. The MS2 site can also be visualized using a locus-specific DNA FISH probe. Unlike YFP-MCP, DNA FISH allows for the detection of the MS2 locus regardless of transcriptional activity, but the approach may not be compatible with all antibodies and likewise requires individual assay optimization.

The MS2 transgene contains a Tet promoter-adjacent array of Lac Operon (LacO) repeats (Janicki et al. 2004). While a LacI repressor is routinely used to tether a protein of interest, such as a fluorescent marker, to LacO arrays, we note that LacI binding poses a block to DNA polymerase progression, resulting in replication stress and a concomitant, MS2-proximal DNA damage response (Beuzer et al 2014; Kim et al 2025, 2018). A similar block is expected for RNA polymerase progression, which may alter repair factor accumulation independent of a given experimental perturbation. When planning to incorporate the LacO/LacI system into the workflow, it is therefore critical to test its impact on the protein of interest both in the presence and absence of Dox induction.

Finally, we note that our system is currently limited to U2OS cells. Studies of other cell lines will require insertion of the original reporter transgene vector (p3216PECMS2β), which may be requested from the authors of the original study (Janicki et al. 2004), along with expression of the YFP-MCP transgene (available from Addgene, e.g. plasmid #101,160**)**. Alternatively, MS2 RNA stem loops may be inserted into introns of endogenous genes through a gene-trap (GT) strategy as recently described (Wan et al 2021). The underlying “Gene Trap—24XMS2 Lentiviral Vector” is available from Addgene (plasmid #174,198).

## Supplementary Information

Below is the link to the electronic supplementary material.

ESM 1 (DOCX 20.7 KB)

## Data Availability

Data is provided within the manuscript or supplementary information files. ImageJ macros for image analysis are available on Github: https://github.com/tguan6/MS2_Enrichment_Profiling.
