# Supplementary material for: Visualizing DNA repair factor recruitment at sites of transcription in single cells
Source: Chromosome Res. 2026 Jan 3;34(1):1. doi: 10.1007/s10577-025-09789-9 (PMC12764657; doi:10.1007/s10577-025-09789-9)
Supplement: Supplementary file 1 — (DOCX 20.7 KB) [file 10577_2025_9789_MOESM1_ESM.docx]

| **REAGENT or RESOURCE** | **SOURCE** | **IDENTIFIER** |
| --- | --- | --- |
| **Primary Antibodies** | | |
| α-TOP1 | Abcam | Cat#ab109374, RRID: AB_10861978 |
| α-XRCC1 | Santa Cruz | Cat#sc-56254, RRID: AB_794191 |
| α-XRCC1 | Novus Biologicals | Cat# NBP1-87154, RRID: AB_11029388 |
| α-TOP1cc | Sigma Aldrich | Cat# MABE1084, RRID: AB_2756354 |
| α-H3K27me3 | Cell Signaling Technologies | Cat#9733,  RRID: AB_2616029 |
| Goat anti-Mouse IgG (H+L) Cross-Adsorbed Secondary Antibody, Alexa Fluor 647 | Invitrogen | Cat#A-21235  RRID: AB_2535804 |
| Goat anti-Rabbit IgG (H+L) Cross-Adsorbed Secondary Antibody, Alexa Fluor 647 | Invitrogen | Cat#A-21244  RRID: AB_2535812 |
| Goat anti-Mouse IgG (H+L) Cross-Absorbed Secondary Antibody, Alex Fluor 594 | Invitrogen | Cat#A-11005,  RRID: AB_2534073 |
| Goat anti-Rabbit IgG (H+L) Cross-Adsorbed Secondary Antibody, Alexa Fluor 594 | Invitrogen | Cat#A-11012,  RRID: AB_2534079 |
| **Chemicals and reagents** | | |
| Camptothecin | Selleckchem | Cat#S1288 |
| Doxycycline hyclate | Sigma-Aldrich | Cat#D9891 |
| 5-Ethynyl-2’-deoxyuridine (EdU) | Sigma-Aldrich | Cat#900584 |
| PDD000172273 (PARGi) | Selleckchem | Cat#S8862 |
| ProLong Dimond Antifade Mountant | Thermo Fisher | Cat# P36965 |
| Hoechst 33342 | Sigma-Aldrich | Cat# 14533 |
| AZDye 647 Azide Plus | Vector Laboratories | Cat#CCT-1482 |
| Paraformaldehyde 20% Solution | Electron Microscopy Sciences | Cat#15713 |
| Poly-L-Lysine Solution | Sigma-Aldrich | Cat#P8920 |
| Dulbecco’s Modified Eagle Medium | Gibco | Cat#11965092 |
| Fetal Bovine Serum | Gemini | Cat#100-106 |
| Bovine Serum Albumin | Sigma-Aldrich | Cat#A2934 |
| TrypLE Express Enzyme | Gibco | Cat#12505010 |
| **Critical commercial assays** | | |
| Click-iT™ Plus EdU Cell Proliferation Kit for Imaging, Alexa Fluor™ 647 dye | Invitrogen | Cat#C10640 |
| Mycoplasma PCR Detection Kit | Abcam | Cat#ab289834 |
| **Experimental models: Cell lines** | | |
| U2OS 2-6-3 | Gift from R. Greenberg, U. Penn | Tang et al, 2013 |
| **Software** | | |
| Zen 3.6 | Zeiss |  |
| ImageJ (FIJI) (2.16.0) | NIH | https://imagej.net/software/fiji/ |
| Prism 10 | GraphPad | https://www.graphpad.com/scientific-software/prism/ |
| **Other** | | |
| LSM900 Airyscan 2 | Zeiss |  |

**Table S1.** Resources used in this study.
